# SGLT2 inhibition for outcomes: Is this the panacea?

**DOI:** 10.1016/j.ahjo.2022.100159

**Published:** 2022-06-28

**Authors:** Keith C. Ferdinand, Neha Arora

**Affiliations:** John W. Deming Department of Medicine, Section of Cardiology, Tulane University School of Medicine, New Orleans, LA, USA

**Keywords:** Sodium-glucose cotransporter-2 inhibitors, Acute myocardial infarction, Heart failure, Health equity, Clinical care improvement

## Abstract

The use of SGLT2 inhibitors, beyond present guideline-directed medical therapy for patients with type 2 diabetes, chronic kidney disease, high atherosclerotic cardiovascular risk and heart failure, may in the future become a significant addition to the treatment of patients with acute myocardial infarction. Ongoing studies, including the PREDOMINANCE trial, may prove beneficial with this class of agents, already demonstrated to improve outcomes in a wide range of patients, with and without type 2 diabetes. Cardiovascular specialists would benefit from increasing their familiarity with this proven therapeutic class in patients with cardiometabolic and cardiovascular disease and increase their utilization in appropriate patients.

In multiple diseases, modern scientists and clinicians have resisted the urge of the ancient siren call to find any **panacea**, named after the Greek goddess of the universal remedy claimed to cure all diseases and prolong life indefinitely. Similar to prior ruminations of the status of statin therapy as a panacea in the pharmacotherapeutic approach to a wide range of cardiovascular diseases (CVD) [1], the use of sodium-glucose cotransporter-2 inhibitors (SGLT2i) may emerge as an unsurpassed, broad intervention in the modern approach to improving outcomes in a wide range of cardiometabolic and CVD states. In consideration of the status of coronary heart disease as the persistent number one cause of death in the U.S. and the unfortunate recent increase in CVD mortality [2], any effort to document additional beneficial evidence-based CVD reduction strategies is laudable.

## 1 Current approaches for acute myocardial infarction

The modern, evidence-based treatment of acute myocardial infarction (AMI) includes acute reperfusion, antiplatelet and antithrombotic treatment, early initiation of renin-angiotensin-aldosterone system inhibition, beta-blockers, and statin therapy. In contemporary practice, in addition to direct percutaneous coronary intervention (PCI), early use of multiple evidence-based medical therapies reduces the risk of adverse cardiac remodeling and development of chronic heart failure (HF), sudden cardiac death, or end-stage heart disease. Patients, with and without type 2 diabetes (T2D), are affected by multiple risk factors which fuel adverse outcomes post AMI. In previous major CVD outcome trials, excluding patients with AMI, at least two SGLT2is have significant cardiorenal and cardiovascular (CV) benefits, and there are also positive outcomes with chronic HF, with both reduced and preserved ejection fraction (HFrEF and HFpEF).

## 2 The proven benefits of SGLT2 inhibitors in cardiovascular disease

In patients with T2D and established atherosclerotic cardiovascular disease (ASCVD), multiple ASCVD risk factors, or diabetic kidney disease, a SGLT2i with demonstrated CV benefit is recommended to reduce the risk of major adverse CVD events and/or HF hospitalizations [3]. Recent guidelines and clinical pathways have confirmed the utility of SGLT2is in a wide range of patients. However, trial evidence has been limited to patients with ASCVD and those with only a remote history of myocardial infarction (MI).

Most patients with T2D also have elevated blood pressure and hypertension (HTN), which is perhaps the most potent and prevalent major risk factor for coronary heart disease. Recent evidence demonstrated significant blood pressure (BP) lowering effects with empagliflozin and other SGLT2is. For instance, in self-identified Black adults with HTN and T2D, empagliflozin has been shown to cause robust BP lowering [4] and significantly reduced 24-hour ambulatory systolic BP versus placebo by Weeks 12 and 24 (placebo-corrected difference, −5.21 mm Hg [95 % CI -9.24, −1.18; P = 0.0117] and − 8.39 mm Hg [95 % CI, −13.74, −3.04; P = 0.0025], respectively). The SGLT2i BP effect was similar to standard antihypertensive monotherapies, suggesting an additional pathway to CVD benefit for this high risk-population.

There is a broad and impressive spectrum of the U.S. Food and Drug Administration's approved indications for SGLT2is (empagliflozin, canagliflozin, dapagliflozin, ertugliflozin) [5]. All four SGLT2is listed have been shown to lower blood glucose levels along with diet and exercise in T2D. Empagliflozin reduces the risk of death from a CV cause in T2D patients with known ASCVD and reduces the risk of CVD death and HF hospitalization in patients with HFpEF and HFrEF. Secondly, canagliflozin reduces the risk of MACE (MI, stroke, and CV death) in T2D patients with known ASCVD and reduces the risk of end-stage renal disease (ESRD), worsening kidney function, CV death, and HF hospitalization in T2D patients with diabetic kidney disease. In addition, dapagliflozin reduces the risk of HF hospitalization in T2D patients with multiple CV risk factors or with known ASCVD and reduces the risk of ESRD, worsening kidney function, CV death, and HF hospitalization in patients with CKD. Dapagliflozin also reduces the risk of CV death and HF hospitalization in patients with HFrEF. The 2022 American Diabetes Association Standards of Care recommend the majority of SGLT2is available in the U.S. (i.e., canagliflozin, dapagliflozin, and empagliflozin) to reduce adverse CV outcomes in adult patients with T2D and established CVD or multiple CV risk factors [6].

## 3 The PREDOMINANCE trial and the potential use of SGLT2 inhibitors in acute myocardial infarction

In this issue of the American Heart Journal (AHJ) Plus: Cardiology Research and Practice, Che et al. describe the design of the provocative PREDOMINACE trial [7]. This randomized, controlled study will examine the potential prevention effects of dapagliflozin on early ventricular dysfunction and remodeling in patients with acute anterior wall ST-segment elevation MI. Despite some basic scientific evidence that SGLT2is can effectively inhibit reperfusion injury after AMI, the clinical utility of SGLT2is in improving reperfusion injury in patients with AMI is unproven. Therefore, the results of this multicenter study in a Chinese population will have profound global implications on the evolving best practices for patients with AMI. Dapagliflozin (10 mg once daily) will be initiated before primary PCI (PPCI) and 30 days after PPCI, and will be compared to a control group. If the final results are positive, SGLT2is will continue to evolve as a class of medications which benefit a wide range of high-risk patients, with and without diabetes. Their use will then extend beyond hyperglycemia, protection against chronic kidney disease (CKD) progression, use in various stages of HF (throughout the range of ejection fractions) and potentially lowering high BP, to treating high-risk patients, with or without remote MI, and acute coronary syndromes.

The financial support of this study from a pharmaceutical company necessitates appropriate independent assessment and replication of the trial findings, and it is somewhat reassuring that the authors have declared that although dapagliflozin is provided free of charge, the investigators are fully responsible for the design and implementation of this study, all research analyses, and the drafting and editing of the paper and its final content [7].

A recent major review describing the potential use of SGLT2is early following an AMI to reduce CV morbidity and mortality by Udell et al. includes possible benefits by attenuation of neurohormonal activation, cardiomyocyte necrosis, and reperfusion injury [5]. If positive results with PREDOMINACE using a SGLT2i in AMI are documented, this may emerge as the newest, and perhaps, most intriguing benefit of this class.

## 4 Prior and additional ongoing trials related to acute myocardial infarction and SGLT2 inhibitors

The AMI multiple adverse outcomes include HF, ventricular arrhythmia and death, and a new agent to assist with risk reduction would be welcomed. While noninvasively assessing cardiac sympathetic and parasympathetic activity using heart rate variability (HRV) and heart rate turbulence (HRT), the recent Effects of empagliflozin versus placebo on cardiac sympathetic activity in acute myocardial infarction patients with type 2 diabetes mellitus (EMBODY) trial was designed to determine whether the SGLT2i improves cardiac nerve activity [8]. A relatively small sample (n = 96) in Japan in a prospective, multicenter, randomized, double-blind, placebo-controlled trial of patients with AMI and T2D received 10-mg empagliflozin or placebo and showed positive changes in the primary endpoint of HRV, HRT, the standard deviation of all 5-min mean normal RR intervals, and the low-frequency-to-high-frequency ratio. This is the first randomized clinical trial to specifically evaluate the effect of empagliflozin on cardiac sympathetic and parasympathetic activity in patients with T2D and AMI.

Currently, in addition to PREDOMINANCE, there are 3 ongoing trials evaluating the efficacy and safety of SGLT2is in AMI: Impact of EMpagliflozin on Cardiac Function and Biomarkers of Heart Failure in Patients with Acute MYocardial Infarction (EMMY); A Streamlined, Multicenter, Randomized, Parallel Group, Double-blind Placebo-controlled Superiority Trial to Evaluate the Effect of EMPAgliflozin on Hospitalization for Heart Failure and Mortality in Patients With aCuTe Myocardial Infarction (EMPACT-MI); and Dapagliflozin Effects on Cardiovascular Events in Patients with an Acute Heart Attack (DAPA-MI) [5].

## 5 Potential use of SGLT2i with left ventricular assist device (LVAD)

In the latest 2022 ACC/AHA Guideline, the use of SGLT-2is among patients with HF, a leading cause of morbidity and mortality globally, is now a Class IA recommendation. The Dapagliflozin and Prevention of Adverse Outcomes in Heart Failure (DAPA-HF) trial and EMPagliflozin outcomE tRial in Patients With chrOnic heaRt Failure With Reduced Ejection Fraction (EMPEROR-Reduced) trial showed the benefit of a SGLT2i (dapagliflozin and empagliflozin, respectively) versus placebo on outcomes in patients with symptomatic HFrEF, reducing the composite of CV death or HF hospitalization by approximately 25 %, irrespective of baseline diabetes status [9]. Furthermore, despite no significant CV mortality benefit with empagliflozin, a meta-analysis of the DAPA-HF and EMPEROR-Reduced trials was associated with a reduction in all-cause mortality and CV death.

The emergence of contemporary durable LVADs in selected patients with New York Heart Association (NYHA) Class IV symptoms, deemed dependent on IV inotropes or temporary mechanical circulatory support, has led to a Class IA guideline recommendation to improve functional status, quality of life, and survival [9]. Nevertheless, the combined benefits of durable mechanical circulatory support and SGLT2is have not been studied. The AHJ Plus has also included a provocative, although non-conclusive, combined retrospective cohort of LVAD patients on SGLT-2is at two major academic centers, showing a good safety profile [10]. This small study of only 33–34 T2D patients, with limited time for follow-up, and stage D HFrEF receiving LVAD support, is stimulating and hypothesis generating. The analysis has very limited power to determine definitively any safety or efficacy signals, particularly without a comparator group, but supports the need for research into the safety and impact of SGLT-2is among LVAD patients.

## 6 The need for health equity in cardiovascular research and application

Unfortunately, despite a higher burden of CVD and T2D among certain racial/ethnic populations in the U.S., such as Hispanic and non-Hispanic Black adults, and a higher burden of cardiometabolic disease, especially in older women and individuals with lower socioeconomic status (SES), the use of SGLT2is among patients with T2D in the U.S. is not applied equitably.

A retrospective cohort study of 934,737 commercially insured T2D adult patients in the U.S. from October 1, 2015, to June 30, 2019, including those with HFrEF, ASCVD, or CKD, confirmed underutilization in certain patient groups [11]. Although the frequency of SGLT2i use increased, this class remained underutilized among patients with HF, CKD, and CVD and there was a significant underutilization associated with the Black race and female sex [11]. Moreover, a higher median household income demonstrated higher rates of SGLT2i utilization.

The intersection of being a Black female and of low-income status may portend widening gaps in longevity and optimal health, even as future studies confirm these novel agents work across the wide spectrum of CVD, including AMI. If SGLT2is are found to benefit patients with AMI, it is important to consider whether a patient has the ability to sustain treatment if initiated in the hospital, taking into account prescription abandonment impacted by economic barriers.

The social determinants of health affect 80 % of chronic disease burden, and only 20 % of outcomes are changed by medical interventions [12]. Therefore, if the unequal use of SGLT2i treatment is not addressed correctly, disparities among patients with cardiometabolic disease, CVD and CKD may worsen in the U.S. Advances in medical care, if not applied equitably to all high risk patients, may have an unintended consequence of widening unaddressed disparities in U.S. CV and cardiometabolic outcomes.

Unfortunately, both women and racially/ethnically diverse populations are often underrepresented in major CV clinical trials impacted by barriers such as fear, mistrust, unawareness about on-going trials, not being approached for participation, and overly restrictive eligibility criteria [13]. The final data from the PREDOMINANCE trial, completed in China, would benefit from a more diverse population so to apply the findings with confidence in the heterogenous U.S. patient population.

## 7 The appropriate use of SGLT2 inhibitors by cardiovascular specialists

Regardless of results in patients with AMI or newer areas of study, CV specialists must increase their knowledge and confidence in the use of SGLT2is in patients, with and without T2D, with established CVD or at high CV risk. Unfortunately, unacceptable real-world data from 2017 to 2018 revealed that only 6 % of eligible patients receive SGLT-2is in clinical practice, and less than 5 % of current prescriptions of SGLT-2is appear to be initiated by cardiologists [14], [15].

The final appropriate application of guideline-directed use of SGLT2is, with or without post AMI outcome studies, must be addressed. In the future, SGLT2i utilization may be a significant leap forward in the armamentarium of the modern treatment of patients suffering an AMI, regardless of diabetic status. However, in the interim, the maximum utility of SGLT2is cannot be realized until the U.S. healthcare system improves insurance and formulary accessibility, and cardiologists increase use to indicated patient populations, regardless of race/ethnicity, sex, gender, and SES.

## Declaration of competing interest

Neha Arora has no disclosures. Dr. Ferdinand is a consultant for Amgen, Sanofi, Boehringer Ingelheim, Novartis, Quantum Genomics, Janssen, Eli Lilly and the principal investigator for Healthy Heart Community Prevention Project.
